# Impact of *TP53* mutations on survival outcomes in the CAR-T era of large B-cell lymphoma

**DOI:** 10.3389/fimmu.2026.1823432

**Published:** 2026-06-03

**Authors:** Rui Liu, Zhonghua Fu, Fan Yang, Miaomiao Cao, Lixia Ma, Yuelu Guo, Biping Deng, Qinlong Zheng, Chen Chen, Bingxin Jiang, Alex H. Chang, Danyang Li, Xiaoyan Ke, Kai Hu

**Affiliations:** 1 Department of Lymphoma and Myeloma Research Center, Beijing GoBroad Hospital, Beijing, China; 2 Cytology Laboratory, Beijing GoBroad Boren Hospital, Beijing, China; 3 Department of Biostatistics, GoBroad Research Center, Beijing, China; 4 Engineering Research Center of Gene Technology, Ministry of Education, Institute of Genetics, School of Life Sciences, Fudan University, Shanghai, China; 5 Shanghai YaKe Biotechnology Ltd., Shanghai, China

**Keywords:** TP53 mutations, large B-cell lymphoma, CAR-T therapy, survival outcomes, prognosis, diffuse large B-cell lymphoma, genomic alterations, next-generation sequencing

## Abstract

**Purpose:**

*TP53* mutations are among the strongest adverse prognostic factors in large B-cell lymphoma (LBCL) treated with chemoimmunotherapy. Whether this unfavorable prognostic impact persists in the era of chimeric antigen receptor (CAR) T-cell therapy remains controversial, largely due to the lack of non–CAR-T comparator cohorts. We aimed to explore whether the adverse prognostic impact of TP53 mutations differs according to treatment modality, particularly in patients receiving CAR-T therapy versus chemotherapy-based approaches.

**Patients and methods:**

We retrospectively analyzed 195 adult patients with r/r LBCL who underwent Next-Generation Sequencing (NGS). *TP53* mutations were identified in 75 patients (38%). Overall, 151 patients received CD19-directed CAR-T cell therapy, while 44 received non–CAR-T chemotherapy-based treatments. Mutational architecture, including mutation type (missense vs. disruptive) and protein domain (DNA-binding domain [DBD] vs. non-DBD), was also evaluated within the TP53-mutant cohort. Patients were stratified into four groups according to *TP53* mutation status and treatment modality. Treatment responses were assessed per Lugano 2014 criteria. Overall survival (OS) and progression-free survival (PFS) were estimated using the Kaplan–Meier method and compared using log-rank tests.

**Results:**

Among *TP53*-mutated patients, CAR-T therapy was associated with significantly improved outcomes compared with chemotherapy, with higher objective response rates (56.6% vs 18.2%) and markedly prolonged median OS (12.13 vs 2.20 months; P <.0001). In *TP53* wild-type patients, CAR-T therapy also resulted in superior survival compared with chemotherapy (median OS, 25.15 vs 8.28 months; P = .003). Within the CAR-T–treated cohort, *TP53*-mutated patients achieved objective and complete response rates comparable to those of *TP53* wild-type patients, although median OS and PFS were shorter. The superior efficacy of CAR-T was consistent across different TP53 mutation types, although patients with mutations localized outside the DBD (non-DBD) demonstrated a trend toward further improved survival (*P* = 0.046). The incidence and severity of CAR-T–related toxicities, including cytokine release syndrome, immune effector cell–associated neurotoxicity syndrome, and grade ≥3 hematologic adverse events, were similar regardless of *TP53* mutation status.

**Conclusion:**

Although *TP53* mutations are associated with poor prognosis in patients treated with chemotherapy, their adverse prognostic impact appears to be attenuated in the context of CAR-T cell therapy. These findings suggest that CAR-T therapy may partially mitigate the negative impact of *TP53* alterations.

## Background

*TP53* alterations have long been recognized as one of the most robust adverse prognostic factors in aggressive B-cell lymphomas (1–3). In the immunochemotherapy era, large cohort studies consistently demonstrated that *TP53* mutations were associated with significantly inferior progression-free survival (PFS) and overall survival (OS) in patients with diffuse large B-cell lymphoma (DLBCL) treated with R-CHOP–based regimens (4). These findings were subsequently confirmed in independent cohorts and reinforced by meta-analyses across multiple non-Hodgkin lymphoma subtypes (1, 2, 5), establishing *TP53* as a stable high-risk molecular marker in the chemotherapy era. Biologically, *TP53* plays a central role in maintaining genomic integrity, and its disruption provides a mechanistic basis for treatment resistance. Loss-of-function *TP53* mutations impair DNA damage response, cell-cycle control, and apoptosis, leading to genomic instability and accelerated clonal evolution (6). Evolutionary and genomic studies further suggest that *TP53*-altered clones may be preferentially selected under therapeutic pressure, contributing to disease relapse and refractory behavior (7). Accordingly, in the relapsed or refractory (r/r) setting, *TP53* alterations may reflect not only adverse prognosis but also a more complex underlying tumor biology.

With the introduction of CD19-directed chimeric antigen receptor T-cell (CAR-T) therapy, the prognostic relevance of *TP53* alterations has been re-examined but remains incompletely defined. Several retrospective studies have suggested that *TP53* genomic alterations may continue to be associated with inferior outcomes following CAR-T therapy, including lower response rates or shorter duration of response (8–10).In addition, whole-genome sequencing analyses have implicated *TP53* alterations as components of complex genomic architectures associated with CAR-T treatment failure (11). However, substantial heterogeneity exists across studies with respect to patient populations, prior lines of therapy, analytical approaches, and follow-up duration, resulting in inconsistent conclusions regarding the prognostic impact of *TP53* alterations in the CAR-T setting.

Notably, most available evidence is derived from single-arm retrospective CAR-T cohorts, often lacking contemporaneous non–CAR-T–treated comparator groups, which limits assessment of whether *TP53* alterations retain independent prognostic significance in the CAR-T era. Moreover, *TP53* alterations frequently co-occur with other high-risk genomic abnormalities, and their role within broader mutational landscapes—particularly in relation to treatment response and resistance following CAR-T therapy—has not been systematically evaluated. Recent systematic reviews and meta-analyses have further highlighted the marked heterogeneity of outcomes among *TP53*-mutated patients treated with CAR-T therapy, suggesting that prognostic effects may be influenced by treatment context, baseline disease characteristics, and co-occurring genomic features (12). To address these gaps, we conducted a real-world study including a contemporaneous non–CAR-T comparator cohort of patients with relapsed or refractory large B-cell lymphoma. By stratifying patients according to *TP53* mutation status and treatment modality, we were able to directly assess whether CAR-T therapy can mitigate the historically adverse prognostic impact of *TP53* alterations. We systematically compared treatment response, survival outcomes, and treatment-related toxicities between CAR-T and chemotherapy-based strategies, and further explored the genomic landscape associated with *TP53* alterations.

## Methods

### Study population

We retrospectively reviewed adult patients (≥18 years) with relapsed or refractory large B-cell lymphoma (r/r LBCL) at Beijing Gobroad Hospital. All patients underwent next-generation sequencing (NGS) either at diagnosis or before salvage therapy. A total of 195 patients with available NGS data were identified. Among them, 75 patients (38%) harbored *TP53* mutations, while 120 patients (62%) had *TP53* wild-type disease.

**Figure 1:**
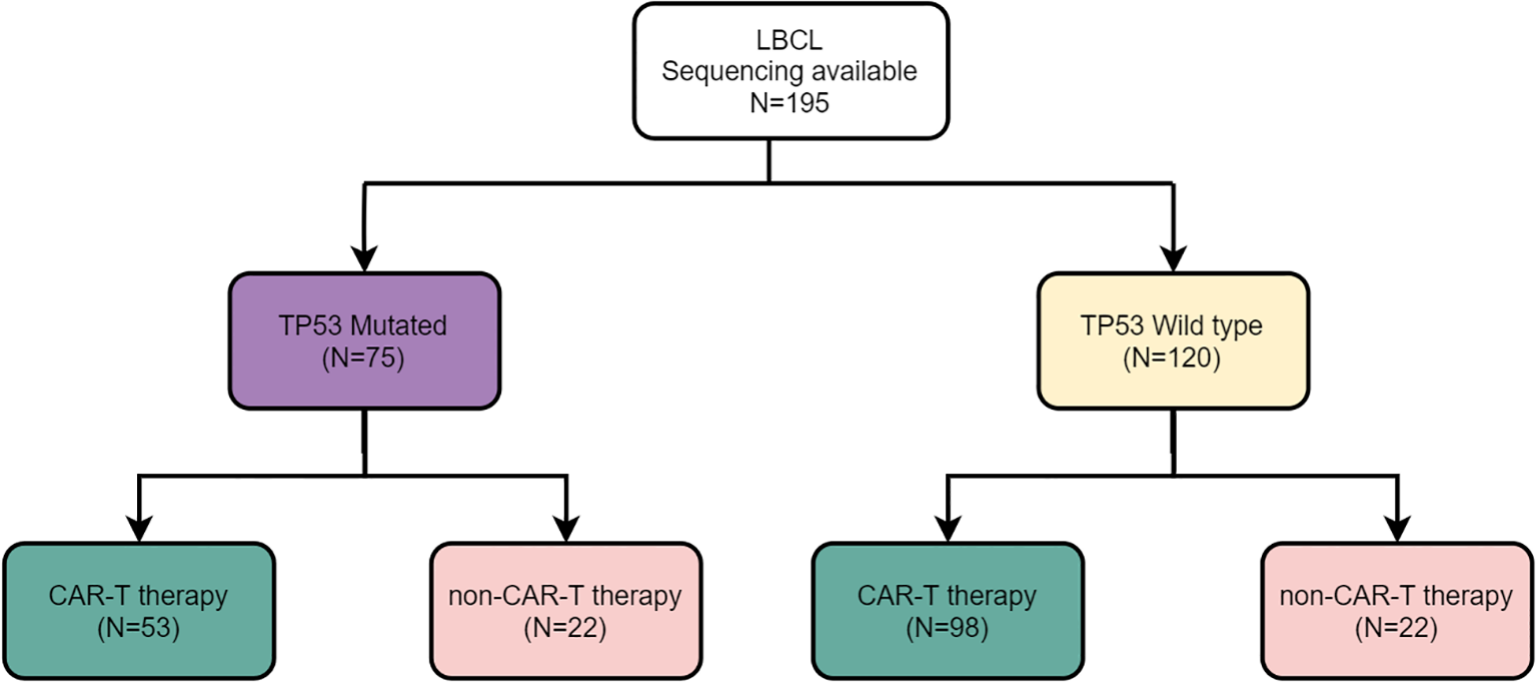
Flow diagram of the study cohort. A total of 195 patients with relapsed or refractory large B-cell lymphoma who underwent tumor genomic sequencing were included and stratified according to *TP53* mutation status and treatment modality.

Overall, 151 patients (77%) received CD19-directed chimeric antigen receptor (CAR) T-cell therapy, while the remaining patients were treated with non–CAR-T, chemotherapy-based salvage regimens. Treatment decisions (CAR-T vs. non–CAR-T) were made based on clinical judgment, patient condition, and treatment accessibility in real-world practice. According to *TP53* mutation status and treatment modality, patients were categorized into four groups: *TP53*-mutated patients treated with CAR-T therapy (n = 53); *TP53*-mutated patients treated with non–CAR-T therapy (n = 22); *TP53* wild-type patients treated with CAR-T therapy (n = 98); and *TP53* wild-type patients treated with non–CAR-T therapy (n = 22) (**Figure 1**).

Baseline demographic, clinical, and molecular characteristics of the study population are summarized in **Table 1**. The study was approved by the Ethics Committee of Beijing Gobroad Hospital. All patients who received CAR-T therapy were enrolled in a registered clinical trial evaluating a CD19-directed CAR-T cell product manufactured by Shanghai YaKe Biotechnology Co., Ltd. (ChiCTR1900020980). The CAR construct was encoded by a lentiviral vector and contained a 4-1BB costimulatory domain and a CD3z signaling domain. The manufacturing process and success rate have been previously described (13). Written informed consent was obtained from all participants or their legal representatives prior to sample collection and treatment. The data cut-off date for this analysis was March 13, 2025.

**Table 1:** Patient characteristics.

Characteristic	TP53mut+CART	TP53mut+nonCART	p_mut_subgroup	TP53wt+CART	TP53wt+nonCART	p_wt_subgroup
Patients, n	53	22		98	22	
Age, median (IQR)	50.0 (41.0–56.0)	55.5 (42.2–70.0)	0.073	55.5 (42.2–63.0)	57.5 (42.0–64.0)	0.546
AGE>60, n (%)	9 (17.0%)	10 (45.5%)	**0.018**	32 (32.7%)	9 (40.9%)	0.466
SEX, male, n (%)	28 (52.8%)	15 (68.2%)	0.306	45 (45.9%)	7 (31.8%)	0.246
Histology, n (%)			0.743			0.050
DLBCL	39 (73.6%)	17 (77.3%)		88 (89.8%)	20 (90.9%)	
HGBL	9 (17.0%)	3 (13.6%)		9 (9.2%)	0 (0.0%)	
PMBL	2 (3.8%)	0 (0.0%)		1 (1.0%)	1 (4.5%)	
TFL	3 (5.7%)	2 (9.1%)		0 (0.0%)	1 (4.5%)	
IPI, n (%)			0.064			0.002
<3	24 (45.3%)	6 (27.3%)		52 (53.1%)	3 (13.6%)	
unknown	3 (5.7%)	5 (22.7%)		5 (5.1%)	4 (18.2%)	
≥3	26 (49.1%)	11 (50.0%)		41 (41.8%)	15 (68.2%)	
Stage, III-IV, n (%)	50 (94.3%)	20 (90.9%)	0.627	85 (86.7%)	22 (100.0%)	0.123
Coo, n (%)			1.000			<0.001
GCB	21 (39.6%)	9 (40.9%)		34 (34.7%)	4 (18.2%)	
Non-GCB	32 (60.4%)	13 (59.1%)		64 (65.3%)	15 (68.2%)	
unknown	0 (0.0%)	0 (0.0%)		0 (0.0%)	3 (13.6%)	
ECOG, ≥2, n (%)	21 (39.6%)	12 (54.5%)	0.309	31 (31.6%)	11 (50.0%)	0.138
Double-hit or triple-hit, n (%)			0.825			0.338
No	34 (64.2%)	15 (68.2%)		65 (66.3%)	17 (77.3%)	
Yes	6 (11.3%)	3 (13.6%)		8 (8.2%)	0 (0.0%)	
unknown	13 (24.5%)	4 (18.2%)		25 (25.5%)	5 (22.7%)	
prior_ASCT, Yes, n (%)	8 (15.1%)	0 (0.0%)	0.096	15 (15.3%)	2 (9.1%)	0.735
refractory to first-line therapy, Yes, n (%)	18 (34.0%)	19 (86.4%)	<0.001	20 (20.4%)	11 (50.0%)	0.007
Early relapse within 6 months, Yes, n (%)	34 (64.2%)	20 (90.9%)	**0.023**	43 (43.9%)	11 (50.0%)	0.641
Early relapse within 12 months, Yes, n (%)	45 (84.9%)	21 (95.5%)	0.268	75 (76.5%)	14 (63.6%)	0.280

The bold values indicate statistically significant differences (P < 0.05).

### Next-generation sequencing

Tumor samples (FFPE or fine-needle aspirates) from 195 patients underwent targeted NGS using a 170-gene B-cell lymphoma panel (**Supplementary Table 1**; Beijing Gobroad Boren Hospital Laboratory). Hybrid capture-based enrichment was performed on exonic regions, achieving >99% coverage with a mean depth >1500×. Somatic variants (SNVs, InDels ≤20 bp) were called using standard pipelines with a variant allele frequency (VAF) >5% and population frequency <1% (gnomAD/ExAC). Variants were annotated against multiple clinical and cancer databases, including COSMIC, ClinVar, and dbSNP. The complete landscape of gene mutations identified across the cohort is detailed in **Supplementary Table 6**.

*TP53* mutation status was defined by nonsynonymous variants, including missense, nonsense, frameshift, and splice-site alterations. Copy number alterations (e.g., 17p deletion) were excluded. *TP53* mutations were further sub-categorized by:1.Mutation type: missense versus disruptive (nonsense, frameshift, or splice-site alterations). 2. Functional domain: the DNA-binding domain (DBD, codons 102–292, exons 5–8) versus non-DBD regions (codons 1–101 and 293–393) (14).

Tumor mutation burden (TMB) was calculated as nonsynonymous variants per Mb. Detailed mutational profiles for all *TP53*-mutated patients, including specific codons and VAFs, are provided in **Supplementary Table 5**.

### Treatment and response assessment

CAR-T cell therapy was administered according to the protocols specified in the registered clinical trial. Non–CAR-T therapy consisted of investigator-selected chemotherapy-based salvage regimens administered at our center.

Treatment response was assessed according to the Lugano 2014 criteria (15). Overall response rate (ORR) was defined as the proportion of patients achieving complete response (CR) or partial response (PR), and complete response rate (CRR) was defined as the proportion of patients achieving CR.

### Study endpoints

The primary study endpoints were ORR, CRR, overall survival (OS), progression-free survival (PFS), and treatment-related adverse events. OS was defined as the time from initiation of treatment to death from any cause or last follow-up. For patients treated with CAR-T therapy, OS was calculated from the date of CAR-T cell infusion. For patients in the non–CAR-T group, OS was calculated from the start date of salvage therapy, in order to ensure comparability between treatment groups. This definition was used to ensure comparability of survival endpoints between treatment groups. PFS was defined as the time from treatment initiation to documented disease progression or death due to lymphoma, whichever occurred first. For CAR-T–treated patients, PFS was measured from the date of CAR-T infusion.

Exploratory endpoints included the impact of *TP53* mutation status and selected clinical and molecular covariates on clinical outcomes.

### Statistical analysis

Continuous variables were summarized as medians with interquartile ranges (IQRs) and compared using the Mann–Whitney U test. Categorical variables were summarized as frequencies and percentages and compared using the χ² test or Fisher’s exact test, as appropriate.

Survival outcomes were estimated using the Kaplan–Meier method and compared between groups using the log-rank test. Univariate and multivariate Cox proportional hazards regression models were used to evaluate prognostic factors for OS and PFS. Variables with a p-value < 0.05 in univariate analyses or those considered clinically relevant were included in multivariate models. The proportional hazards assumption was evaluated using the Schoenfeld residuals test, and no violation of the assumption was observed. An oncoplot was generated to illustrate the mutation profiles of the top 50 most frequently mutated genes across the 194 tumor samples. All statistical analyses were performed using R4.5.2 and Python (version 3.14) with the lifelines library for advanced survival modeling and subgroup subclassification analysis. All tests were two-sided, and a p-value < 0.05 was considered statistically significant.

## Results

### Patient characteristics

The study cohort comprised 195 adult patients with r/r LBCL, with a median age of 54 years (range 15–80). TP53 mutations were identified in 75 patients (38%). Overall, the population exhibited high-risk features: 91% had advanced-stage disease, 48% had an IPI score ≥ 3, and 38% had an ECOG performance status ≥ 2. Most patients (52%) were heavily pretreated with ≥ 3 prior lines of therapy, and 34% were refractory to first-line treatment. Detailed baseline characteristics stratified by TP53 status and treatment modality are presented in **Table 1** and **Supplementary Table 2**. While clinical features were largely comparable across groups, significant imbalances were observed within the *TP53*-mutant stratum. Specifically, patients in the non-CAR-T group were significantly older (age > 60: 45.5% vs. 17.0%; *P* = 0.018) and more likely to be refractory to first-line therapy (86.4% vs. 34.0%; *P* < 0.001) or experience early relapse within 6 months (90.9% vs. 64.2%; *P* = 0.023) compared to the CAR-T group. In the *TP53* wild-type stratum, the non-CAR-T cohort also demonstrated a higher prevalence of IPI ≥ 3 (*P* = 0.002) and distinct cell-of-origin distributions (*P* < 0.001). These observed imbalances highlight a clinical selection bias where patients with more aggressive or treatment-refractory disease were predominantly represented in the non-CAR-T cohort.

### Response and survival outcomes by *TP53* status and treatment

Patients were stratified into four subgroups according to *TP53* mutation status and treatment modality (CAR-T vs. non–CAR-T), and their baseline characteristics are summarized in **Table 1**. Among patients with *TP53* mutations, CAR-T therapy achieved a markedly higher objective response rate (ORR) compared with chemotherapy (56.6% [30/53] vs. 18.2% [4/22]). Median overall survival (OS) was significantly longer in the CAR-T group than in the non–CAR-T group (12.13 vs. 2.20 months, *P* < 0.0001).

**Figure 2:**
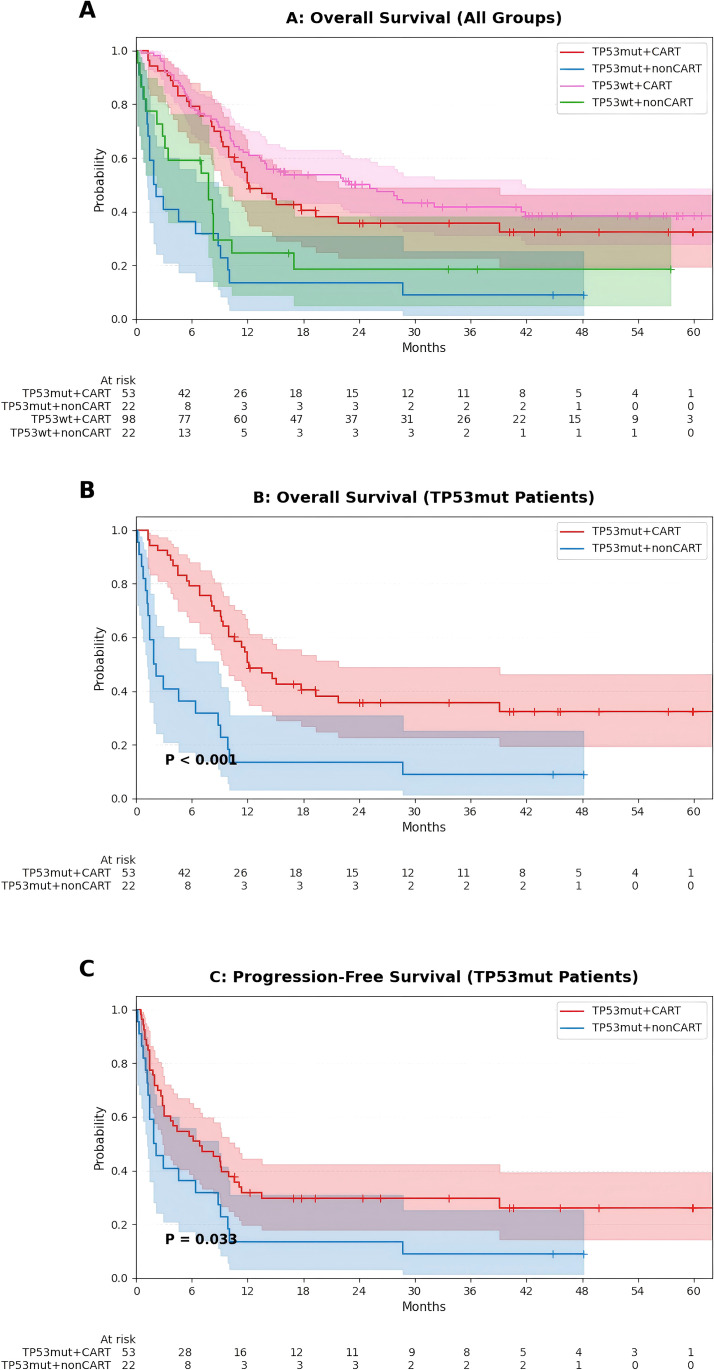
Survival outcomes based on *TP53* status and CAR-T therapy. **(A)** Kaplan-Meier analysis of overall survival (OS) for the entire cohort, stratified into four groups: *TP53*-mutated patients treated with CAR-T (*TP53* mutated+CART), *TP53*-mutated patients not treated with CAR-T (*TP53* mutated+nonCART), TP53-wild-type patients treated with CAR-T (*TP53* wild-type+CART), and *TP53* wild-type patients not treated with CAR-T (*TP53* wild-type +nonCART). **(B)** Subgroup analysis of OS specifically within the *TP53*-mutated patient group, comparing those treated with CAR-T versus non-CAR-T. **(C)** Subgroup analysis of progression-free survival (PFS) specifically within the *TP53*-mutated patient group, comparing those treated with CAR-T versus non-CAR-T. P values were calculated using the log-rank test. The tables below each plot indicate the number of patients at risk at different time points.

Similarly, in patients with *TP53* wild-type disease, CAR-T therapy was associated with superior outcomes. The ORR was 45.9% (45/98) in the CAR-T group versus 13.6% (3/22) in the non–CAR-T group. Median OS was 25.15 months in CAR-T–treated patients compared with 8.28 months in those who did not receive CAR-T therapy (*P* = 0.0030) (**Figure 2**). Overall, clinical characteristics across the four subgroups were generally comparable, although some imbalances were observed (**Table 1**).

To assess the impact of subsequent therapies on survival, we analyzed the management of patients who progressed after CAR-T therapy (n=61). The post-progression treatments were heterogeneous, including repeat CAR-T therapy (n=35), CAR-T combined with ASCT (n=16), and others (**Supplementary Table 4**). Notably, patients with mutations tended to receive less intensive therapies compared to wild-type patients, often due to rapid clinical deterioration after the initial CAR-T failure.

### Outcomes of CAR-T therapy according to *TP53* mutation status

A total of 151 patients received CAR-T cell infusion, including 53 patients with *TP53* mutations and 98 patients with *TP53* wild-type disease. The median age of CAR-T–treated patients was 53 years, and 73 patients (48.3%) were male. The median infusion dose was 1.35 (range 0.01-8) × 10^6/kg. Among *TP53*-mutant patients treated with CAR-T therapy, the ORR was 56.6% (30/53), with a complete response rate (CRR) of 39.6% (21/53). The median OS was 12.13 months (95% CI, 8.337–15.926), and the median progression-free survival (PFS) was 6.87 months (95% CI, 1.745–11.997).

In *TP53* wild-type patients receiving CAR-T therapy, the ORR was 45.9% (45/98), and the CRR was 35.7% (35/98). Median OS was 25.15 months (95% CI, 12.543–37.758), and median PFS was 8.98 months (95% CI, 5.087–12.864). Efficacy outcomes of CAR-T therapy according to *TP53* mutation status are summarized in **Table 2**.

**Table 2:** Clinical efficacy of CAR-T cell therapy according to *TP53* mutation status.

*TP53* status	CAR-T therapy (n=151)			
	ORR (n, %)	CRR (n, %)	Median OS, months (95%,CI)	Median PFS, months (95%,CI)
*TP53*-mutant (N = 53)	30 (56.6)	21 (39.6)	12.13 (8.34-15.93)	6.87 (1.75-12.00)
*TP53* wild-type (N = 98)	45 (45.9)	35 (35.7)	25.15 (12.54-37.76)	8.98 (5.09-12.86)

Notably, the relatively wide 95% confidence intervals observed in the *TP53*-mutant group suggest a degree of statistical uncertainty, which may reflect the limited sample size and underlying heterogeneity of this subgroup, and therefore warrant cautious interpretation.

### Prognostic factors for survival: univariate and multivariable analyses

**Figure 3:**
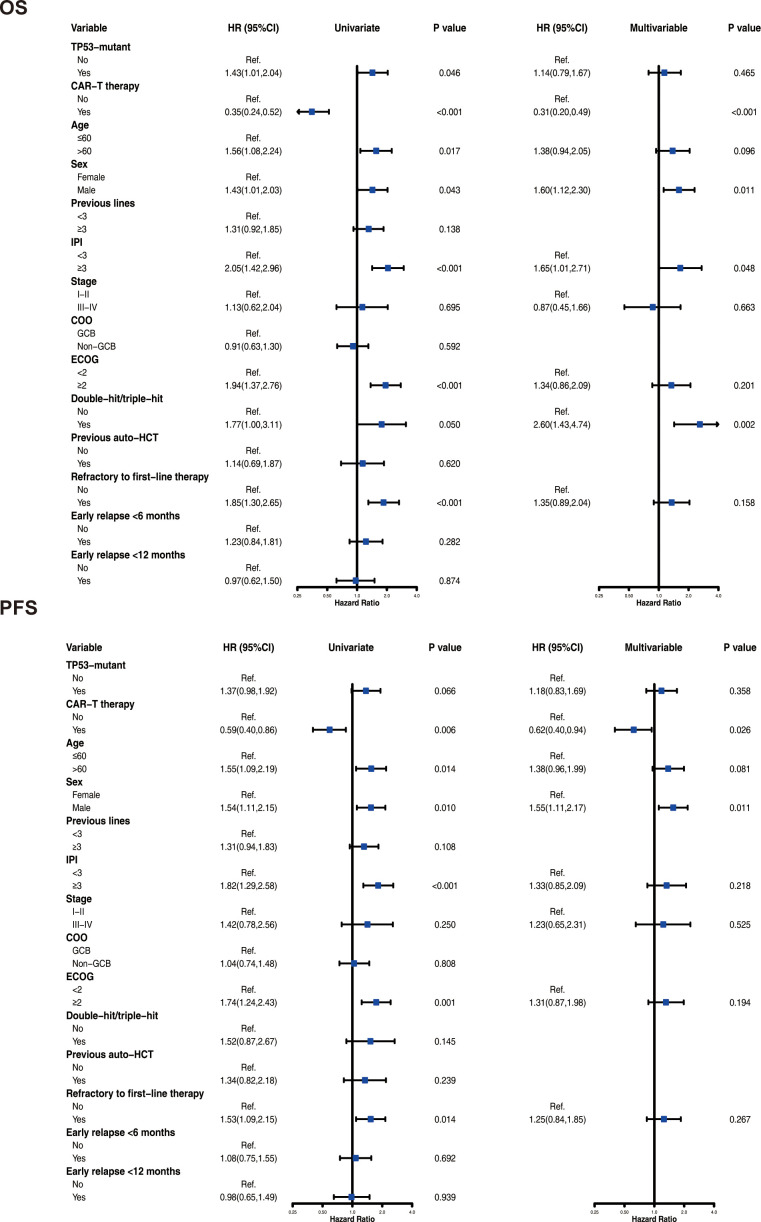
Prognostic factors for overall survival (OS)/progression-free survival (PFS). Hazard ratios (HRs) and 95% confidence intervals (CIs) for clinical and genetic variables are shown. The analysis was performed using a Cox proportional hazards model. Variables with a P value < 0.1 in the univariate analysis were included in the multivariate model. The blue squares represent the HR, and the horizontal lines indicate the 95% CI.

Prognostic Factors for SurvivalTo evaluate whether the survival benefit of CAR-T therapy was independent of baseline imbalances, we performed univariate and multivariable Cox regression analyses (**Figure 3**). In the multivariable model for OS, which adjusted for TP53 status, age, IPI score, sex, and double-hit/triple-hit status, CAR-T therapy emerged as a potent independent protective factor (HR = 0.31; 95% CI: 0.20–0.49; P < 0.001). Conversely, male sex (HR = 1.61; P = 0.011) and double-hit/triple-hit status (HR = 2.60; P = 0.002) were identified as independent risk factors for OS. Consistent results were observed for PFS, where CAR-T therapy remained independently associated with superior outcomes (HR = 0.62; 95% CI: 0.40–0.94; P = 0.026). Notably, while TP53 mutation status was an adverse prognostic factor for OS in univariate analysis (P = 0.046), its significance was attenuated in the multivariable model (P = 0.465). This finding suggests that the robust therapeutic efficacy of CAR-T cell therapy may partially mitigate the negative prognostic impact historically associated with TP53 alterations in the chemoimmunotherapy era.

### Safety and toxicity of CAR-T therapy

The safety profile of CAR-T therapy within 30 days after infusion was comparable between *TP53*-mutant and *TP53* wild-type patients (**Table 3**). Hematologic toxicities were nearly universal in both groups. Neutropenia of any grade occurred in 53 patients (100%) with *TP53* mutations and 97 patients (99%) with *TP53* wild-type disease; grade ≥3 neutropenia was observed in 49 patients (92%) and 52 patients (98%), respectively. Thrombocytopenia of any grade occurred in 49 patients (92%) versus 90 patients (92%), with grade ≥3 thrombocytopenia in 40 patients (75%) and 65 patients (66%). Anemia of any grade was observed in all patients, with grade ≥3 anemia reported in 36 patients (68%) with *TP53* mutations and 64 patients (65%) with *TP53* wild-type disease.

**Table 3:** Safety profile within 0–30 days after CAR-T infusion.

Adverse event	*TP53*-mutant (n=53)	*TP53*–wild-type (n=98)	*p*
Hemotologic toxicity			
Neutropenia, any grade	53 (100%)	97 (99%)	1.00
Grade ≥3	52 (98%)	92 (94%)	0.422
Thrombocytopenia, any grade	49 (92%)	90 (92%)	1.00
Grade ≥3	40 (75%)	65 (66%)	0.271
Anemia, any grade	53 (100%)	98 (100%)	1.00
Grade ≥3	36 (68%)	64 (65%)	0.857
Hematologic recovery (median days, range)			
Neutrophils	14 (6–NR)	15.5 (3–NR)	0.485
Platelets	11.5 (7–NR)	11 (3–NR)	0.822
Hemoglobin	15 (9–NR)	15 (5–NR)	0.950
Infections			
Bacterial infection	15(28%)	24(24%)	0.698
Fungal infection	7(13%)	9(9%)	0.580
Viral infection	2(4%)	4(4%)	1.00
CAR-T Specific Toxicity			
CRS, any grade	42 (79%)	81 (83%)	0.663
Grade ≥3	5 (9%)	7 (7%)	0.754
ICANS, any grade	4 (8%)	5 (5%)	0.720
Grade ≥3	4 (8%)	3 (3%)	0.241
Management			
Tocilizumab use	5(9%)	7(7%)	0.754
Corticosteroid use	27(51%)	41(42%)	0.316

NR, not reached; CRS, cytokine release syndrome; ICANS, immune effector cell-associated neurotoxicity syndrome.

Rates of bacterial, fungal, and viral infections were similar between the *TP53*-mutant and *TP53* wild-type cohorts (28% vs. 24%, 13% vs. 9%, and 4% vs. 4%, respectively).

Hematologic recovery following CAR-T infusion was also comparable between groups. Median time to neutrophil recovery was 14 days (range, 6–not reached [NR]) in *TP53*-mutant patients and 15.5 days (range, 3–NR) in *TP53* wild-type patients. Median platelet recovery times were 11.5 days (range, 7–NR) and 11 days (range, 3–NR), respectively, while median time to hemoglobin recovery was 15 days in both groups.

Cytokine release syndrome (CRS) occurred in 42 patients (79%) with *TP53* mutations and 81 patients (83%) with *TP53* wild-type disease. Grade ≥3 CRS was reported in 5 patients (9%) and 7 patients (7%), respectively. Median time to CRS onset was 0.88 days (range, 0.03–11.53) in the *TP53*-mutant group and 1.27 days (range, 0.02–15.23) in the *TP53* wild-type group, with median CRS durations of 7.67 days and 6 days, respectively. Immune effector cell–associated neurotoxicity syndrome (ICANS) was uncommon, occurring in 4 patients (8%) with *TP53* mutations and 5 patients (5%) with *TP53* wild-type disease; grade ≥3 ICANS was reported in 4 patients (8%) and 3 patients (3%), respectively. Use of tocilizumab and corticosteroids was similar between groups (9% vs. 7% and 47% vs. 42%, respectively).

### Genomic landscape and impact of *TP53* mutational determinants

**Figure 4:**
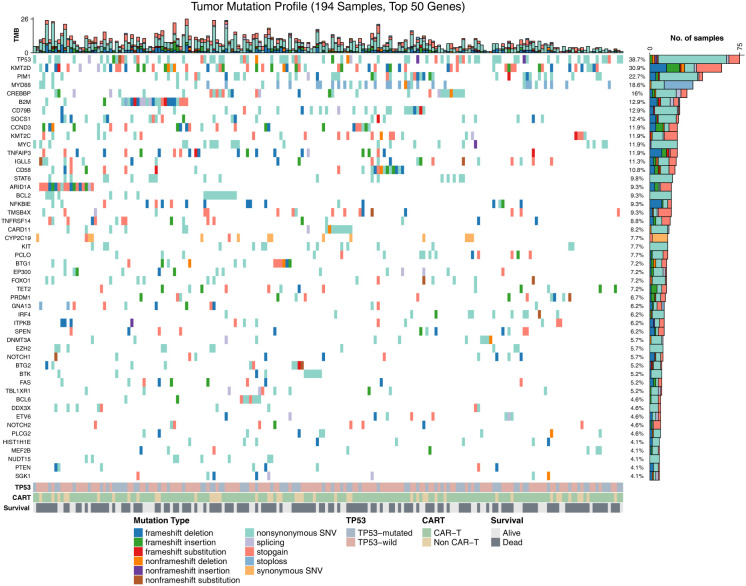
Genomic landscape of the patient cohort. The oncoprint displays the top 50 most frequently mutated genes. Each column represents an individual patient sample, and each row represents a gene. Different colors correspond to different mutation types as indicated in the legend. The upper bar plot shows the tumor mutation burden (TMB) for each sample. The right bar plot summarizes the mutation frequency of each gene across the entire cohort. The bottom panels display clinical annotations including treatment groups, best overall response (BOR), and survival status. Each column represents an individual patient.

To characterize the genomic landscape of our cohort, we analyzed the mutation profiles of 194 tumor samples (one patient lacked complete variant-level information, including mutation type and allelic frequency, and was therefore excluded from visualization). The overall tumor mutation burden (TMB) varied considerably across the cohort. The top 50 most frequently mutated genes are depicted in a comprehensive oncoprint (**Figure 4**). The most prevalent somatic mutation was found in the tumor suppressor gene *TP53*, occurring in 38.7% (75/194) of the samples. This was followed by mutations in genes related to epigenetic regulation, including *KMT2D* (30.9%) and *CREBBP* (16.0%). Key genes involved in signaling pathways, such as *PIM1* (22.7%), *MYD88* (18.6%), and *B2M* (12.9%), also exhibited high mutation frequencies. The majority of identified mutations were nonsynonymous single-nucleotide variants (SNVs), with functionally significant alterations like frameshift deletions and stop gain mutations also being recurrently observed in critical genes like *TP53* and *KMT2D*. This mutational signature highlights the genetic heterogeneity of the disease and underscores the central role of aberrant tumor suppression, epigenetic modification, and signaling pathways in its pathogenesis. To allow for visual correlation between genetic alterations and clinical outcomes, we annotated the figure with clinical tracks including treatment group (CAR-T vs. Non-CAR-T), best clinical response (CR, PR, SD, or PD), and final survival status (Alive vs. Dead).

### Genomic determinants of outcome: mutation type vs. protein domain

**Figure 5:**
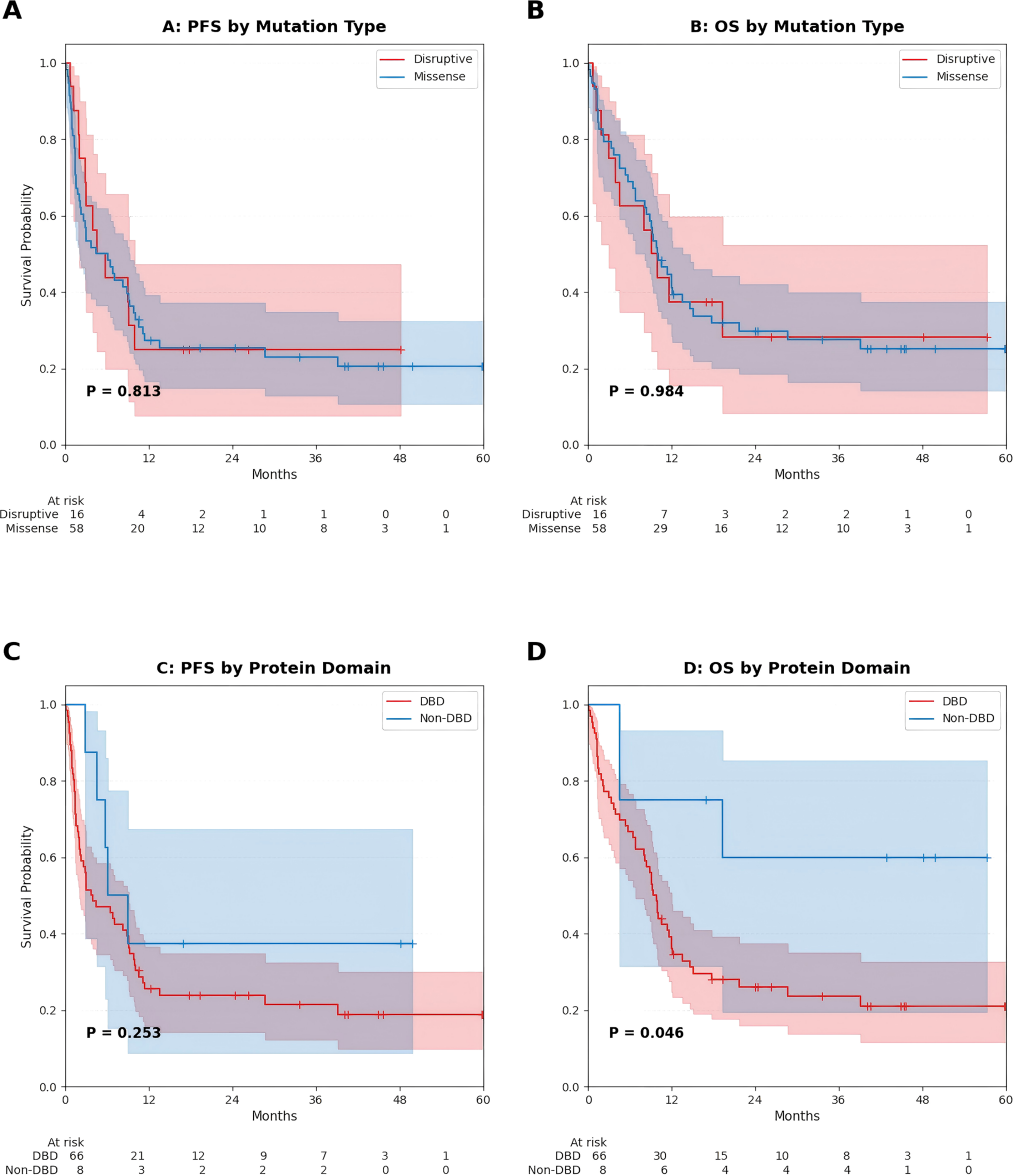
Survival outcomes of TP53-mutated LBCL patients by molecular subclassification (n=74).Kaplan–Meier analysis of progression-free survival (PFS) and overall survival (OS) stratified by **(A, B)** mutation type (missense vs. disruptive) and **(C, D)** protein domain [DNA-binding domain (DBD) vs. non-DBD]. While mutation type showed no prognostic impact (P > 0.05), non-DBD mutations were associated with significantly improved OS compared to DBD mutations (median OS: NR vs. 9.86 months; P = 0.046). P-values were determined by the log-rank test. Tick marks indicate censored data. HR, hazard ratio; NR, not reached.

Among the 74 TP53-mutated patients analyzed, 89% (66/74) harbored mutations within the DBD, while 11% (8/74) had mutations in the non-DBD regions. The most frequent DBD mutations were located in exons 5, 7, and 8, consistent with known hotspot patterns in LBCL. Survival was analyzed according to *TP53* mutation type (missense vs. disruptive) and protein domain (DBD vs. non-DBD). Kaplan–Meier analysis demonstrated no significant differences in PFS or OS according to mutation type (median PFS: 6.12 vs. 4.57 months, *P* = 0.929; median OS: 10.08 vs. 9.07 months, P = 0.818; **Figures 5A, B**). In contrast, mutations located outside the DNA-binding domain (non-DBD) were associated with significantly improved OS (median OS: NR vs. 9.86 months, *P* = 0.046; **Figure 5D**), although the PFS difference did not reach statistical significance (median PFS: 9.04 vs. 4.01 months, *P* = 0.253; **Figure 5C**). Multivariate Cox proportional hazards analysis confirmed these findings. The *TP53* protein domain (non-DBD vs. DBD) was significantly associated with a reduced risk of death (OS HR = 0.26, 95% CI 0.08–0.90, P = 0.03), whereas mutation type alone showed no significant effect on PFS or OS. These results suggest that protein domain location captures the functional impact of *TP53* mutations more accurately than the simple mutation type.

### Genomic and histological determinants of outcome

In parallel, we evaluated the influence of histological heterogeneity within the *TP53*-mutant cohort (n=75). Patients with high-grade B-cell lymphoma (HGBL, n=12), confirmed by FISH for MYC and BCL2/BCL6 rearrangements (double-hit/triple-hit), demonstrated a trend toward inferior survival compared to those with other LBCL subtypes (Other, n=63). The median PFS for the HGBL and Other groups was 6.44 months (95% CI: 2.86–9.07) and 1.51 months (95% CI: 0.99–9.19), respectively (*P* = 0.22; **Supplementary Figure 1A**). Notably, a marginal difference was observed in overall survival (OS), with the HGBL group exhibiting a shorter median OS of 4.54 months (95% CI: 1.45–11.38) compared to 10.59 months (95% CI: 8.84–15.12) in the Other group (P = 0.087; **Supplementary Figure 1B**). Cox proportional hazards analysis further suggested that the HGBL subtype may carry an increased risk of death (HR = 1.82 [1/0.55], *P* = 0.09). These findings indicate that the clinical outcome of *TP53*-mutated LBCL is modulated by histological aggressiveness.

## Discussion

Pivotal trials such as ZUMA-1, JULIET and TRANSCEND have established CD19-directed CAR-T therapy as an effective treatment for Relapsed/Refractory LBCL, yielding durable responses even in high-risk subgroups (16–18). Our study extends these findings by demonstrating that the survival benefit of CAR-T persists even in patients with *TP53* mutations when compared with a contemporaneous chemotherapy cohort. By including a non–CAR-T comparator group, we were able to directly assess the extent to which CAR-T therapy mitigates the adverse prognostic impact of *TP53* alterations, which has remained controversial in prior single-arm studies with limited (8, 10, 11, 19, 20).

*TP53* mutations are traditionally associated with chemoresistance and inferior survival in aggressive B-cell lymphomas (3, 12). Biologically, *TP53* mutations promote chemoresistance by disrupting p53-dependent DNA damage responses, including apoptosis and cell cycle arrest, thereby enabling malignant cells to evade chemotherapy-induced cell death. This mechanism has been consistently associated with inferior treatment outcomes (21–23). Consistent with prior literature, *TP53*-mutated patients receiving conventional chemotherapy in our cohort had markedly poor outcomes (median OS 2.2 months).

CAR-T–associated toxicities were comparable between *TP53*-mutant and wild-type patients, with similar incidences of CRS, ICANS, hematologic toxicities, and hematopoietic recovery times. These findings indicate that *TP53* mutation status does not confer additional safety risk, supporting the clinical feasibility of CAR-T therapy in high-risk molecular subgroups (18, 24).

Our genomic analysis confirmed *TP53* as the most frequently mutated gene (38.7%), alongside recurrent alterations in epigenetic regulators (*KMT2D, CREBBP)* and signaling pathway components(*MYD88, CD79B*). The high prevalence of mutations in epigenetic modifiers, particularly KMT2D (30.9%) and CREBBP (16.0%), aligns with the established understanding that dysregulation of the epigenome is a fundamental pathogenic mechanism in this malignancy (25–28). The frequent mutations in genes associated with B-cell receptor (BCR) and NF-κB signaling, such as *MYD88* and *CD79B*, further corroborate the importance of these pathways as core drivers of cell survival and proliferation. Interestingly, while the overall mutation profile of our cohort was largely consistent with prior reports, we observed a relatively high frequency of *PIM1* mutations (22.7%). *PIM1* kinase is involved in *JAK/STAT* and can interact with *MYC (*29, 30). However, the clinical significance of these PIM1 mutations in our cohort remains unclear. Give the exploratory nature of this finding, it should be interpreted cautiously as hypothesis-generating, and further studies are required to further studies are required to determine whether PIM1 mutations interact with TP53 status to further modulate treatment resistance.

Our findings underscore the prognostic heterogeneity of *TP53* mutations beyond a binary ‘mutant vs. wild-type’ classification. Specifically, the observed survival advantage in patients with non-DBD mutations suggests that the structural site of the mutation may more accurately reflect functional impairment than the biochemical type (missense vs. disruptive). This is consistent with recent evidence from Yu et al. (2025) (31), who highlighted that specific motifs within the DBD, such as Loop-L2, Loop-L3, and LSH, are critical drivers of poor prognosis.

Furthermore, the inclusion of HGBL (double-hit/triple-hit) status adds another layer of complexity. While our subgroup analysis was limited by sample size, the trend toward worse outcomes in *TP53*-mutated HGBL patients aligns with the hypothesis that genomic instability and high-grade morphology may synergistically confer resistance to therapy. Collectively, these data support the move toward a more granular risk stratification in LBCL, integrating both molecular topography and histological subtypes to optimize clinical decision-making in the CAR-T era.

In our cohort, the overall response rate (ORR) of patients treated with the non-commercial CAR-T product (56.6% and 45.9%) appeared lower than that reported in pivotal trials such as ZUMA-1. This difference likely reflects the higher baseline risk of our study population, including a greater proportion of patients with poor performance status, high IPI scores, and early relapse or refractory disease, rather than intrinsic differences in CAR-T efficacy.

Several limitations of this study should be acknowledged. The retrospective, single-center design introduces inherent selection bias. Treatment allocation was not randomized; patients in the non-CAR-T cohort frequently presented with higher age, higher IPI scores, and a significantly greater prevalence of primary refractory disease. As noted in **Table 1**, the non-CAR-T group often represented patients with rapidly progressive disease who were ineligible for or unable to access cellular therapy. This imbalance likely contributed to the poor outcomes in the non-CAR-T group and may have exaggerated the survival benefit of CAR-T therapy.

However, it is noteworthy that the survival advantage of CAR-T therapy remained highly significant (HR = 0.31 for OS) even after rigorous adjustment for these baseline imbalances and *TP53* status in our multivariable model. Interestingly, while *TP53* mutation was a strong negative predictor in the chemotherapy era, its prognostic weight was reduced in our multivariable analysis, suggesting that CAR-T cell therapy may possess the biological capacity to bypass traditional p53-mediated chemoresistance pathways. While our findings are encouraging, the small size of the non-CAR-T cohort and the heterogeneity of post-progression treatments mean these results should be interpreted as hypothesis-generating. Prospective, multicenter studies are required to confirm whether CAR-T therapy can truly “level the playing field” for patients with high-risk *TP53* mutations.

In addition, the relatively small sample size of the non–CAR-T cohort limited the feasibility of applying propensity score matching or other adjustment methods. Post-progression treatments following CAR-T therapy were heterogeneous, including repeat CAR-T infusion, stem cell transplantation, and other salvage approaches. While these subsequent interventions could potentially confound the overall survival (OS) analysis (**Supplementary Table 4****),** the significant survival advantage observed in the CAR-T group—even after adjusting for clinical baseline factors in our multivariate model—suggests that the primary treatment modality remains a decisive factor in improving outcomes for patients with *TP53-*mutated LBCL.

Finally, cytogenetic abnormalities, including MYC and BCL2 rearrangements, were not systematically assessed in a substantial proportion of patients due to the lack of routine FISH testing in real-world practice. This limitation restricted our ability to fully evaluate their prognostic impact and may have introduced additional bias. Prospective studies with standardized molecular assessments and larger sample sizes are needed to validate our findings.

In conclusion, TP53 mutations remain a strong adverse prognostic factor in patients receiving conventional chemotherapy. However, CAR-T therapy substantially attenuates this negative effect, conferring meaningful survival benefits without excess toxicity. These findings support the integration of genomic risk stratification into treatment decision-making for r/r LBCL and suggest that TP53 mutation status should not preclude the use of CAR-T therapy in eligible patients.

## Data Availability

The original contributions presented in the study are included in the article/**Supplementary Material**. Further inquiries can be directed to the corresponding author.

## References

[1] Zenz T Kreuz M Fuge M Klapper W Horn H Staiger AM . TP53 mutation and survival in aggressive B cell lymphoma. Int J Cancer. (2017) 141:1381–8. doi: 10.1002/ijc.30838. PMID: 28614910

[2] Xu P Liu X Ouyang J Chen B . TP53 mutation predicts the poor prognosis of non-Hodgkin lymphomas: Evidence from a meta-analysis. PloS One. (2017) 12:e0174809. doi: 10.1371/journal.pone.0174809. PMID: 28369138 PMC5378372

[3] Landsburg DJ Morrissette JJ Nasta SD Barta SK Schuster SJ Svoboda J . TP53 mutations predict for poor outcomes in patients with newly diagnosed aggressive B-cell lymphomas in the current era. Blood Adv. (2023) 7:7243–53. doi: 10.1182/bloodadvances.2023011384. PMID: 37851898 PMC10698538

[4] Xu-Monette ZY Wu L Visco C Tai YC Tzankov A Liu WM . Mutational profile and prognostic significance of TP53 in diffuse large B-cell lymphoma patients treated with R-CHOP: report from an International DLBCL Rituximab-CHOP Consortium Program Study. Blood. (2012) 120:3986–96. doi: 10.1182/blood-2012-05-433334. PMID: 22955915 PMC3496956

[5] Negara I Tomuleasa C Buruiana S Efremov DG . Molecular subtypes and the role of TP53 in diffuse large B-cell lymphoma and Richter syndrome. Cancers Bsl. (2024) 16:14. doi: 10.3390/cancers16122170. PMID: 38927876 PMC11201917

[6] Chen X Zhang T Su W Dou Z Zhao D Jin X . Mutant p53 in cancer: from molecular mechanism to therapeutic modulation. Cell Death Dis. (2022) 13:974. doi: 10.1038/s41419-022-05408-1. PMID: 36400749 PMC9674619

[7] Rushton CK Arthur SE Alcaide M Cheung M Jiang A Coyle KM . Genetic and evolutionary patterns of treatment resistance in relapsed B-cell lymphoma. Blood Adv. (2020) 4:2886–98. doi: 10.1182/bloodadvances.2020001696. PMID: 32589730 PMC7362366

[8] Shouval R Alarcon Tomas A Fein JA Flynn JR Markovits E Mayer S . Impact of TP53 genomic alterations in large B-cell lymphoma treated with CD19-chimeric antigen receptor T-cell therapy. J Clin Oncol. (2022) 40:369–81. doi: 10.1200/jco.21.02143. PMID: 34860572 PMC8797602

[9] Porpaczy E Wohlfarth P Königsbrügge O Rabitsch W Skrabs C Staber P . Influence of TP53 mutation on survival of diffuse large B-cell lymphoma in the CAR T-cell era. Cancers Bsl. (2021) 13:10. doi: 10.3390/cancers13225592. PMID: 34830747 PMC8616128

[10] Xue B Liu Y Zhou J Zhou L Ye S Lu Y . CD19 CAR-T treatment shows limited efficacy in r/r DLBCL with double expression and TP53 alterations. Cytotherapy. (2024) 26:1465–71. doi: 10.1016/j.jcyt.2024.07.011. PMID: 39217529

[11] Jain MD Ziccheddu B Coughlin CA Faramand R Griswold AJ Reid KM . Whole-genome sequencing reveals complex genomic features underlying anti-CD19 CAR T-cell treatment failures in lymphoma. Blood. (2022) 140:491–503. doi: 10.1182/blood.2021015008. PMID: 35476848 PMC9353150

[12] Qi W Zhang Y Hao X Yang P Wang J Wu C . Outcomes of wild type and TP53-mutated B cell Malignancy patients receiving CAR-T cell therapy: a systematic review and meta-analysis. J Cell Mol Med. (2025) 29:e70818. doi: 10.1111/jcmm.70818 40984040 PMC12454170

[13] Pan J Yang JF Deng BP Zhao XJ Zhang X Lin YH . High efficacy and safety of low-dose CD19-directed CAR-T cell therapy in 51 refractory or relapsed B acute lymphoblastic leukemia patients. Leukemia. (2017) 31:2587–93. doi: 10.1038/leu.2017.145. PMID: 28490811

[14] Huang M Jin J Zhang F Wu Y Xu C Ying L . Non-disruptive mutation in TP53 DNA-binding domain is a beneficial factor of esophageal squamous cell carcinoma. Ann Transl Med. (2020) 8:316. doi: 10.21037/atm.2020.02.142. PMID: 32355760 PMC7186752

[15] Cheson BD Fisher RI Barrington SF Cavalli F Schwartz LH Zucca E . Recommendations for initial evaluation, staging, and response assessment of Hodgkin and non-Hodgkin lymphoma: the Lugano classification. J Clin Oncol. (2014) 32:3059–68. doi: 10.1200/jco.2013.54.8800. PMID: 25113753 PMC4979083

[16] Locke FL Ghobadi A Jacobson CA Miklos DB Lekakis LJ Oluwole OO . Long-term safety and activity of axicabtagene ciloleucel in refractory large B-cell lymphoma (ZUMA-1): a single-arm, multicentre, phase 1–2 trial. Lancet Oncol. (2019) 20:31–42. doi: 10.1016/s1470-2045(18)30864-7. PMID: 30518502 PMC6733402

[17] Schuster SJ Bishop MR Tam CS Waller EK Borchmann P McGuirk JP . Tisagenlecleucel in adult relapsed or refractory diffuse large B-cell lymphoma. N Engl J Med. (2019) 380:45–56. doi: 10.1056/nejmoa1804980. PMID: 30501490

[18] Abramson JS Palomba ML Gordon LI Lunning MA Wang M Arnason J . Lisocabtagene maraleucel for patients with relapsed or refractory large B-cell lymphomas (TRANSCEND NHL 001): a multicentre seamless design study. Lancet. (2020) 396:839–52. doi: 10.1016/s0140-6736(20)31366-0. PMID: 32888407

[19] Wei J Xiao M Mao Z Wang N Cao Y Xiao Y . Outcome of aggressive B-cell lymphoma with TP53 alterations administered with CAR T-cell cocktail alone or in combination with ASCT. Signal Transduc Tgt Ther. (2022) 7:101. doi: 10.1038/s41392-022-00924-0. PMID: 35399106 PMC8995369

[20] Cai Z Zou D Ma Q Sun W Guo Y . Combined autologous hematopoietic stem cell transplantation and CD19 CAR T-cell therapy for relapsed/refractory diffuse large B-cell lymphoma with TP53 mutation: A case report. SAGE Open Med Case Rep. (2025) 13:2050313X241306236. doi: 10.1177/2050313x241306236. PMID: 40078172 PMC11898238

[21] Wang H Guo M Wei H Chen Y . Targeting p53 pathways: mechanisms, structures, and advances in therapy. Signal Transduc Tgt Ther. (2023) 8:92. doi: 10.1038/s41392-023-01347-1. PMID: 36859359 PMC9977964

[22] Krishnaraj J Yamamoto T Ohki R . p53-dependent cytoprotective mechanisms behind resistance to chemo-radiotherapeutic agents used in cancer treatment. Cancers Bsl. (2023) 15:4. doi: 10.3390/cancers15133399. PMID: 37444509 PMC10341282

[23] Alsulami AF . Mutational disruption of TP53: a structural approach to understanding chemoresistance. Int J Mol Sci. (2025) 26:9. doi: 10.3390/ijms26189135. PMID: 41009698 PMC12470219

[24] Neelapu SS Locke FL Bartlett NL Lekakis LJ Miklos DB Jacobson CA . Axicabtagene ciloleucel CAR T-cell therapy in refractory large B-cell lymphoma. N Engl J Med. (2017) 377:2531–44. doi: 10.1056/nejmoa1707447. PMID: 29226797 PMC5882485

[25] Intlekofer AM Joffe E Batlevi CL Hilden P He J Seshan VE . Integrated DNA/RNA targeted genomic profiling of diffuse large B-cell lymphoma using a clinical assay. Blood Cancer J. (2018) 8:60. doi: 10.1038/s41408-018-0089-0. PMID: 29895903 PMC5997645

[26] Karube K Enjuanes A Dlouhy I Jares P Martin-Garcia D Nadeu F . Integrating genomic alterations in diffuse large B-cell lymphoma identifies new relevant pathways and potential therapeutic targets. Leukemia. (2018) 32:675–84. doi: 10.1182/blood.v128.22.152.152 PMC5843901 28804123

[27] Gao F Tian L Shi H Zheng P Wang J Dong F . Genetic landscape of relapsed and refractory diffuse large B-cell lymphoma: a systemic review and association analysis with next-generation sequencing. Front Genet. (2021) 12:677650. doi: 10.3389/fgene.2021.677650. PMID: 34925435 PMC8675234

[28] Berendsen MR Stevens WBC van den Brand M van Krieken JH Scheijen B . Molecular genetics of relapsed diffuse large B-cell lymphoma: insight into mechanisms of therapy resistance. Cancers Bsl. (2020) 12:11. doi: 10.3390/cancers12123553. PMID: 33260693 PMC7760867

[29] Zhang H Lu Y Zhang T Guan Q Wang X Guo Y . PIM1 genetic alterations associated with distinct molecular profiles, phenotypes and drug responses in diffuse large B-cell lymphoma. Clin Transl Med. (2022) 12:e808. doi: 10.1002/hon.3165_468 35415904 PMC9005929

[30] Ma J Yan Z Zhang J Zhou W Yao Z Wang H . A genetic predictive model for precision treatment of diffuse large B-cell lymphoma with early progression. biomark Res. (2020) 8:33. doi: 10.1186/s40364-020-00214-3. PMID: 32864130 PMC7448459

[31] Yu J He Q Yan Y Liu K Xie R Ma J . Prognostic impact of TP53 mutations in diffuse large B-cell lymphoma. Ann Hematol. (2026) 105:102. doi: 10.1007/s00277-026-06882-9. PMID: 41665720 PMC12891153

