# Supplementary figures and images for: Impact of TP53 mutations on survival outcomes in the CAR-T era of large B-cell lymphoma
Source: Front Immunol. 2026 Jun 3;17:1823432. doi: 10.3389/fimmu.2026.1823432 (PMC13272385; doi:10.3389/fimmu.2026.1823432)

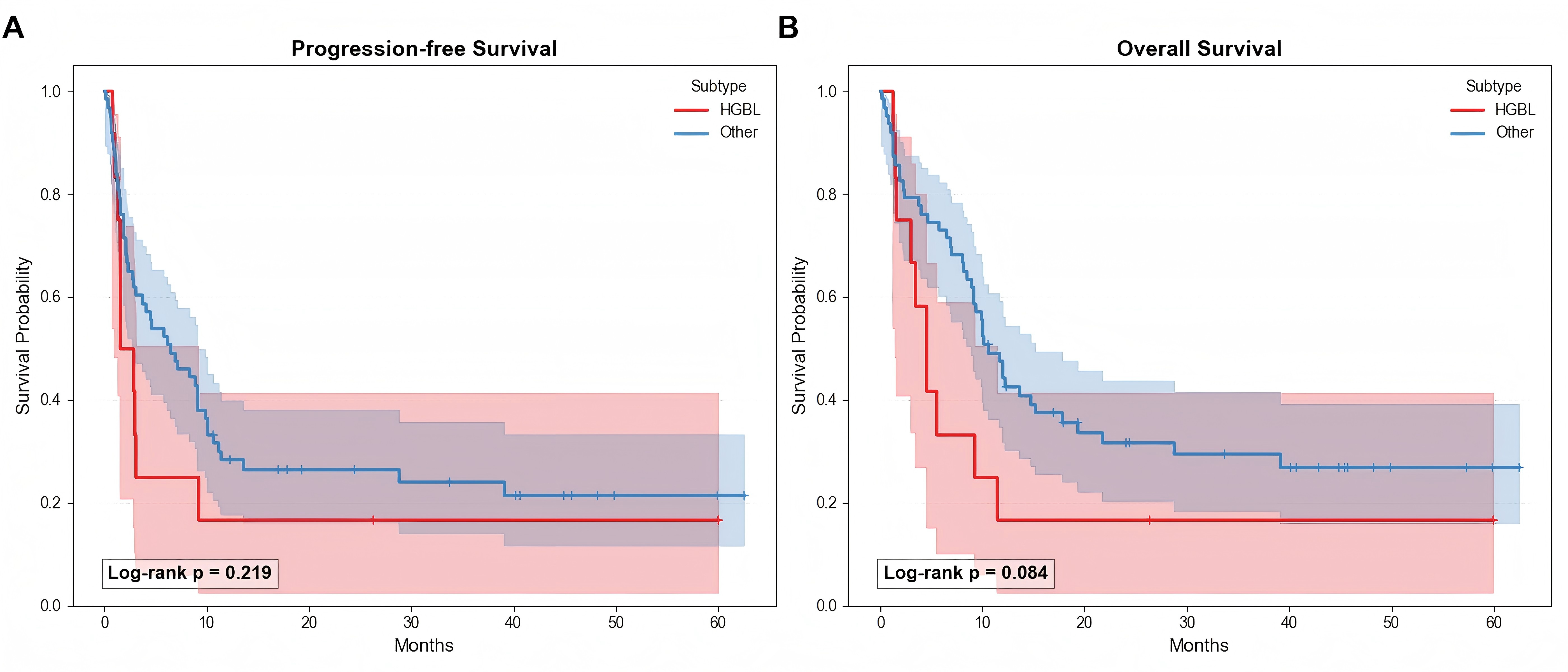

Supplement: Supplementary file 1 [file Image1.jpeg]
